# QSAR Modeling to Predict Aquatic Toxicity Across Multiple Species

**DOI:** 10.3390/toxics14060498

**Published:** 2026-06-07

**Authors:** Iglika Lessigiarska, Petko Alov, Maria Angelova, Stefan Ivanov, Parashkev Katerski, Radostina Nikolova-Kejova, Ilza Pajeva, Tania Pencheva, Ivanka Tsakovska

**Affiliations:** 1 Institute of Biophysics and Biomedical Engineering, Bulgarian Academy of Sciences, Acad. Georgi Bonchev Str., Bl. 21, 1113 Sofia, Bulgaria; petko@biophys.bas.bg (P.A.); maria.angelova@biomed.bas.bg (M.A.); stefan@biophys.bas.bg (S.I.); pkaterski@biomed.bas.bg (P.K.); radost@biomed.bas.bg (R.N.-K.); pajeva@biomed.bas.bg (I.P.); tania.pencheva@biomed.bas.bg (T.P.); 2 Faculty of Pharmacy, Medical University of Sofia, Dunav 2 Str., 1000 Sofia, Bulgaria; 3 Centre of Excellence in Informatics and Information and Communication Technologies, 1113 Sofia, Bulgaria

**Keywords:** QSAR, QSAAR, random forest, *Raphidocelis subcapitata*, *Daphnia magna*, zebrafish embryo, fathead minnow

## Abstract

This study addresses the growing need for efficient and reliable application of New Approach Methodologies (NAMs) to assess aquatic toxicity of chemicals in response to increasing environmental contamination and regulatory demands. Particular emphasis is placed on in silico methods, especially quantitative structure–activity relationship (QSAR) modeling. Curated and structurally diverse datasets were compiled for representative aquatic organisms from different trophic levels, including the microalga *Raphidocelis subcapitata*, the crustacean *Daphnia magna*, and fish species (zebrafish embryo and fathead minnow). The models demonstrated consistently strong predictive performance across the evaluated assays. They were based on interpretable molecular descriptors associated with lipophilicity, polarity, and molecular reactivity. Furthermore, interspecies quantitative structure–activity–activity relationship (QSAAR) models were developed, demonstrating that toxicity data from lower trophic levels, combined with structural descriptors, can effectively predict fish toxicity. These models support cross-species extrapolation and contribute to environmental hazard assessment and regulatory decision-making.

## 1. Introduction

Aquatic ecosystems are increasingly exposed to chemical stressors from industrial, agricultural, and urban sources, resulting in adverse effects on both the environment and human health. Traditionally, experimental ecotoxicological testing is used as the standard approach for assessing chemical hazards. However, the large number of existing and newly synthesized chemicals makes comprehensive in vivo testing impractical. Consequently, regulatory frameworks, such as REACH, encourage the use of New Approach Methodologies (NAMs), including in vitro assays and in silico approaches [1]. Among these, in silico methods are the most straightforward and fastest as they can predict toxicity directly from the chemical structure. In this context, quantitative structure–activity relationship (QSAR) modeling has been established as an effective tool in the field of environmental risk assessment [2].

Aquatic toxicity assessment frequently relies on model organisms representing different trophic levels, including microalgae such as *Raphidocelis subcapitata*, crustaceans such as *Daphnia magna*, and fish species including fathead minnow (*Pimephales promelas*) and zebrafish (*Danio rerio*) [3]. Toxicity responses vary across species due to differences in physiology, metabolic capacity, lifespan, growth rate, reproduction, and development, highlighting the need for both species-specific and integrative predictive models [4].

During the past decade, numerous QSAR models have been developed to predict toxicity toward aquatic organisms. Earlier models were primarily based on linear statistical methods such as multiple linear regression (MLR) and partial least squares regression (PLS), employing physicochemical descriptors related to lipophilicity, polarity, steric, and electronic properties. While some models are restricted to particular chemical classes [5,6,7], other are based on broader chemical space, including diverse chemical structures, usually collected from extensive online databases like ECOTOX [8]. Further, most published models are developed to predict toxicity for a particular organism [9].

Fewer studies provide models for endpoints covering different organisms and different trophic levels [10,11].

More recently, machine learning algorithms, including support vector machines (SVMs), neural networks (NNs), gradient boosting, and random forest (RF), have demonstrated improved capability for modeling nonlinear relationships between chemical structure and biological response. For instance, a QSAR model with reliable statistical performance for predicting EC_10_ toxicity of 334 organic chemicals toward the microalga *Raphidocelis subcapitata* has been reported by Yu [12] using an SVM algorithm. In a further study, RF classification and regression models for the same organism have been derived using quantum-chemical descriptors to represent chemical structures of training (251 compounds) and test data (83 compounds) [13]. One of the largest dataset-based QSAR models has been reported by Aalizadeh et al. [14]. It is built on an experimental acute toxicity dataset (pLC_50_), containing toxicity data for *Daphnia magna* after 48 h of exposure, split into training (1026 compounds), test (327 compounds), and additional evaluation (660) sets. The exhaustive internal and external validation of the SVM model demonstrates its reliability for predicting this endpoint. Further curation of this database has led to the development of RF classification models based on 1517 compounds split into training data (758 compounds) and test data (759 chemicals), with an accuracy of the models in the interval of 88.3–92.3% for the training set and 85.6–87.5% for the test set [15]. As an example of the application of NNs in QSAR ecotoxicity modeling, the study by Wang and Chen [16] reports QSAR models based on a radial basis function (RBF) NN for predicting the acute toxicity of chemicals to fathead minnow (955 compounds) [16]. A hybrid quantum particle swarm optimization algorithm has been employed to jointly optimize model parameters and select key molecular descriptors. Multiple RBF-based QSAR models have been developed, demonstrating strong predictive performance in both cross-validation and external validation. The study has identified the distribution coefficient, molar refractivity, and ionization potential as key factors influencing toxicity. To further improve predictive accuracy, a consensus model has been proposed by combining individual RBF models. The Monte Carlo optimization procedure has been applied in the CORAL software [17] as a tool for building up QSAR models for acute fish embryo toxicity dataset composed of 411 chemicals using SMILES-based descriptors [18]. They have allowed for identification of structural features that significantly influence toxicity. The model’s reliability has been supported by multiple random training/validation splits.

Beyond single-species predictions, there is a persistent interest in developing interspecies QSAAR (quantitative structure–activity–activity relationships) models that leverage data across multiple taxa [19,20,21]. Integrated models can identify structural features that consistently drive toxicity across organisms and facilitate cross-species extrapolation. Such integrative modeling approaches are particularly valuable for regulatory frameworks that aim to minimize animal testing while ensuring environmental safety.

Another important topic in the field is the integration of large curated toxicity databases with computational modeling frameworks. Publicly available data repositories and regulatory databases provide extensive experimental information that can be used to develop robust predictive models. However, the quality and consistency of these datasets are critical for reliable QSAR modeling. Data curation processes, including removal of duplicates, standardization of chemical structures, and verification of experimental conditions, are essential steps in ensuring model reliability and compliance with the OECD principles for QSAR validation [22].

Despite the significant progress in QSAR modeling, several challenges remain in the prediction of aquatic toxicity. One major limitation is the heterogeneity of experimental datasets, which often originate from different laboratories and experimental protocols. Additionally, the structural diversity of environmental contaminants requires models capable of handling complex chemical spaces. Machine learning approaches combined with carefully curated datasets offer a promising strategy to address these challenges by improving predictive performance and expanding the applicability domain of the QSAR models.

In this context, the present study focuses on the development of predictive QSAR models for aquatic toxicity using RF regression and RF classification algorithms. The RF method has been selected because of several advantages: the models obtained are robust toward noisy and heterogeneous datasets, they can effectively handle large descriptor numbers, and are less prone to overfitting compared to many other machine learning methods. These characteristics make RF especially suitable for modeling structurally diverse environmental chemicals and complex toxicological endpoints [13]. Models are developed for *Raphidocelis subcapitata*, *Daphnia magna*, fathead minnow, and zebrafish embryo, and extended to interspecies models that integrate multi-taxa data. By combining species-specific and cross-species modeling strategies, the research seeks to develop interpretable, descriptor-transparent predictive models and provide a robust computational framework for predicting chemical toxicity in aquatic ecosystems.

## 2. Materials and Methods

### 2.1. Toxicity and Structural Data

Aquatic toxicity endpoints towards the following species were included in the investigation:

- *Raphidocelis subcapitata*—half-maximum inhibitory concentrations (E_r_C_50_) according to the OECD Guideline for the Testing of Chemicals No 201 “Freshwater Alga and Cyanobacteria, Growth Inhibition Test” [23]. The guideline recommends the use of EC_50_ calculated from inhibition of the algal growth rate (E_r_C_50_) at 72 h exposure period.
- *Daphnia magna*—half-maximum effective concentrations (EC_50_) according to the OECD Guideline for the Testing of Chemicals No 202 “*Daphnia* sp. Acute Immobilisation Test” [24]. The guiding recommends the use of EC_50_ calculated from immobilization, recorded after 48 h exposure to the test substance of young daphnids.
- *Danio rerio* (zebrafish embryo)—half-maximum lethal concentration (LC_50_) to fish embryo according to the OECD Guideline for the Testing of Chemicals No 236 “Fish Embryo Acute Toxicity (FET) Test” [25].
- *Pimephales promelas* (fish fathead minnow)—half-maximum lethal concentration (LC_50_) at 96 h exposure period according to the OECD Guideline for the Testing of Chemicals No 203 “Fish, Acute Toxicity Testing” [26].

As already discussed, data curation represents a fundamental aspect of computational toxicology studies. This issue has been extensively addressed in the chemoinformatics literature, including the recent review by Esaki and Ikeda [27], which highlights that variability in assay conditions, experimental protocols, and laboratory practices constitutes a major challenge for the development of reliable QSAR models. To address this issue, careful dataset curation procedures were applied in the present study. In particular, single assays corresponding to the OECD Test Guidelines were deliberately selected, as they are widely used for regulatory purposes. This choice was also motivated by the intention to comply with OECD QSAR Validation Principle 1, namely the requirement for a clearly defined endpoint [22]. Data were collected from multiple sources, including a large number of structurally diverse compounds belonging to different chemical classes and with various industrial applications (summarized in Table 1). Detailed structural and toxicological data are presented in the Supplementary QSAR data file (File S1). If reported, data for chemicals and active ingredients with purity > 95%, were used. Inorganic compounds, metal complexes, and mixtures were not included in the dataset. In case the toxicity was reported as exceeding a given value, that value was used as a conservative estimate of a possible worst-case situation. In case of multiple data for the same compound, the average value of the reported toxicities from different sources was used; compounds in which the ratio between the maximum and the minimum toxicity values was greater than 3 were removed in accordance with the threshold established in [28].

The collected toxicity concentrations were transformed to mmol/L, and afterwards to negative decimal logarithm, log(1/half-maximum toxicity concentration) (pEC_50_, pE_r_C_50_, pLC_50_). This transformation is used in the QSAR modeling as the toxicity occurs through intermolecular interactions and the effect is linearly related to the logarithm of the molar concentration.

### 2.2. Descriptors

PaDEL version 2.18 [51] was used to calculate 2D molecular descriptors. The “Remove Salt” option was applied to exclude salts from the calculations, resulting in the generation of 756 possible descriptors per structure. They include logP (octanol–water partition coefficients), specific atom, bond and group counts, hydrogen bond (H-bond) donor and acceptor counts, molar refractivity, descriptors of molecular size and shape, connectivity and topological descriptors, and electrotopological states.

Descriptors without variance and with more than 99% zero values were removed from the datasets. The descriptor values were normalized before the QSAR model derivation.

### 2.3. Development of QSAR and Classification Models

The datasets were divided into training (75%) and test (25%) sets by ranking the toxicity endpoint values and selecting every fourth compound for the test set, thereby ensuring even distribution of toxicity values across both sets.

In order to remove compounds with high leverage in the descriptor space, hat statistics [52] was used. The hat matrix H is calculated as follows (X is the matrix representation of the descriptor space, X^T^ is its transpose):

H = X(X^T^X)^−1^X^T^ (1)

The hat value of a chemical in the descriptor space is the corresponding diagonal element h_ii_ of the hat matrix:

h_ii_ = x_i_^T^(X^T^X)^−1^x_i_ (2)

where x_i_ is the descriptor row-vector of the compound. If a compound has a hat value greater than 3(p + 1)/n (where p is the number of model variables, and n is the number of the compounds in the set) it is assessed as possessing high leverage on the dataset and is removed from the data.

In the current study, compounds with residuals of prediction in the target (toxicity) space with more than 2.5σ (standard deviation of the residuals) were excluded from the training set of the final model in accordance with the outliers’ estimation proposed by Gramatica et al. [32].

Random forest regression (RFR) and random forest classification (RFC) were used to develop the QSAR regression and classification models [53]. They were executed using RandomForestRegressor [54] and RandomForestClassifier [55] from sklearn library in Python 3.14.4 [56]. The number of trees (n_estimators) was set to 500. The maximal number of levels (max_depth) was 10 and the minimum node size (min_samples_leaf) was 5.

The goodness-of-fit of the RFR models was assessed by the coefficient of determination (R^2^), the adjusted coefficient of determination (R^2^_adj_) and the standard error of estimate (SEE) (see Supplementary statistical data file (File S3)).

Internal validation by the leave-one-out (LOO) coefficient of determination (Q^2^) and the out-of-bag (OOB) coefficient (R^2^_oob) was performed. The OOB statistic is obtained during the bootstrapping procedure and is calculated from the predicted values of samples that are not included in the bootstrap training sample. The error estimated by the OOB procedure is almost identical to that obtained by cross-validation [57]. The concordance correlation coefficient (CCC) was used to assess the concordance between the observed and predicted values of the test set (see Supplementary statistical data file (File S3)).

The best-subset approach for descriptor selection was applied using in-house Python script. Due to its extensive computational needs, it was applied in two steps. During the first step all possible models with one and two descriptors were generated, and around 40 descriptors from the models with the best prediction performance on the test set (assessed by test R^2^) were selected. In the second step, the best-subset approach was applied to the descriptors selected during the first step. Models were run by including up to 20 descriptors. The limitations of 40 for the descriptors in the first step and 20 in the second step were chosen as a trade-off between the need for extensive exploration of the descriptor space and the long computational time required by the procedure. Descriptors that intercorrelated with absolute values of the pair intercorrelation coefficients R of more than 0.7 were not included together in the same model. The two best-performing models per endpoint were selected by the values of R^2^_oob and the test R^2^.

In order to minimize the impact of the randomness in the RF regression and RF classification approaches, 10 runs for each model were performed. The average values of the statistical parameters were reported, along with the ranges across the 10 runs to assess model stability.

The Shapley additive explanations (SHAP) values [58] were used to evaluate the descriptor’s importance for the predicted toxicity endpoint. SHAP values were calculated for each compound and for each descriptor in the model. Their sum for a given compound is equal to the difference between its predicted value and the average model prediction for the set. In order to assess the importance of a given descriptor in the model, the absolute SHAP values averaged over all compounds were used. The positive or negative impact of the descriptors on the endpoint were estimated based on the sign of the correlation between the descriptor’s values and its SHAP values. The SHAP values were calculated using the shap library of Python 3.14.4 [59].

To develop classification models, the boundaries for toxicity classification were set according to Annex I in reference [60]. According to the Regulation, substances for which adequate chronic toxicity data are not available are classified using data for 96 h LC_50_ for fish, 48 h EC_50_ for crustaceans, or 72 or 96 h E_r_C_50_ for algae or other aquatic plants. The following classification boundaries are adopted: category Chronic 1 includes chemicals with values of 96 h LC_50_ for fish, 48 h EC_50_ for crustaceans, or 72 or 96 h E_r_C_50_ for algae or other aquatic plants, smaller than 1 mg/L; chemicals are classified under category Chronic 2 if the above endpoints are >1 to ≤10 mg/L; substances are classified under category Chronic 3 if the above endpoints are >10 to ≤100 mg/L.

In order to transfer the above boundaries into mmol/L units, the mean molecular weight of all compounds investigated in this study was calculated, and the value of 227.8 g/mol was obtained. Thus, 0.0439 mmol/L corresponding to the boundary of 10 mg/L and 0.439 mmol/L corresponding to the boundary of 100 mg/L were used in the study to classify the compounds. The classification is presented in Table 2. Compounds with endpoint values of less than 0.0439 mmol/L (corresponding to Chronic categories 1 and 2) were classified in class 1 (labeled as “Toxic” in the following text). Compounds with endpoint values of between 0.0439 mmol/L and 0.439 mmol/L (corresponding to Chronic category 3) were classified in class 2 (labeled as “Harmful”), and compounds with endpoint values of above 0.439 mmol/L were classified in class 3 (labeled as “Non-toxic”).

The classification models were assessed according to accuracy (Acc) and the quadratic weighted Cohen’s Kappa (qCK) statistics [61] (see Supplementary statistical data file (File S3)).

The percentage of correct classifications was compared to the percentage of cases that would have been correctly classified by chance alone (see Supplementary statistical data file (File S3)).

## 3. Results

### 3.1. Summary of the Toxicity and Structural Data Used for Modeling

In Table 1, the main characteristics of the collected datasets used for the development of the QSAR models are provided, including: the toxicity endpoints used for the selected aquatic organisms; the number of compounds included in the training and test sets, and the ranges (in negative decimal logarithm, units mmol/L) of the compounds’ toxicity. The scientific literature and databases from more than 25 available sources were used for data collection.

**Figure 1:**
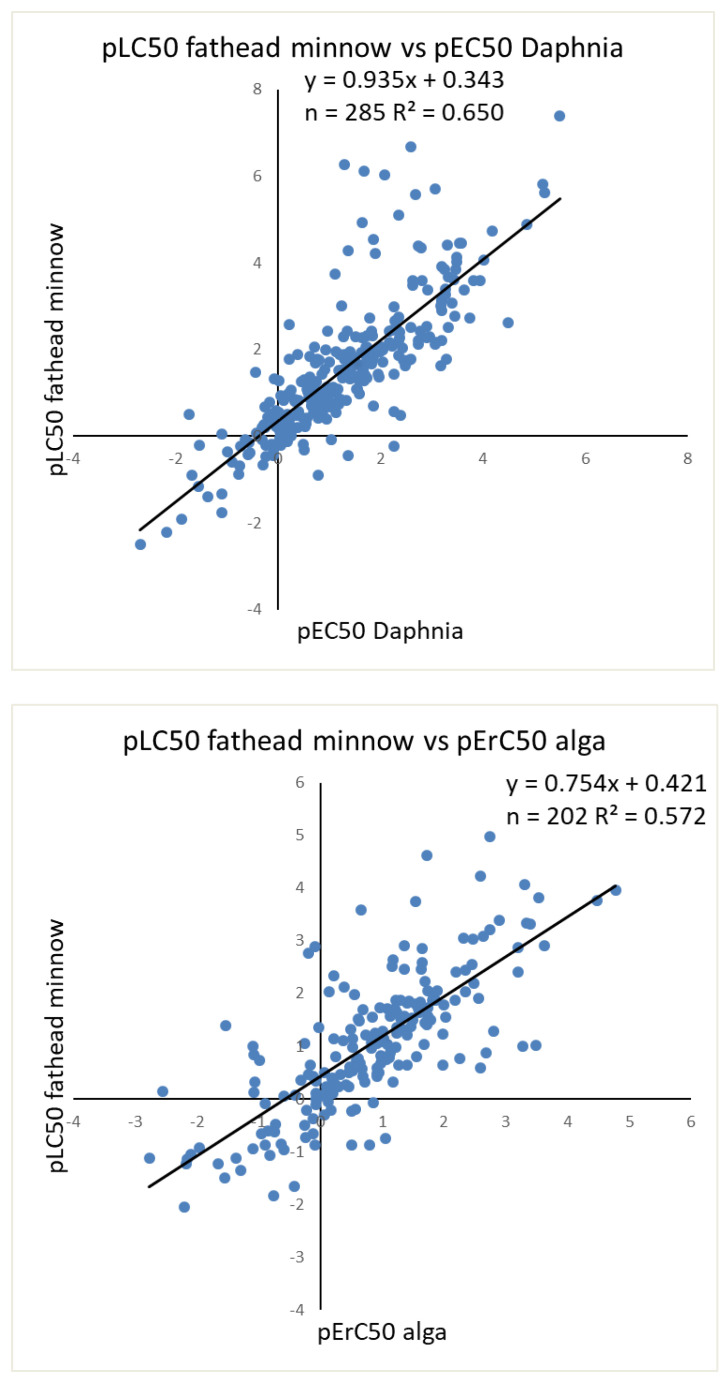

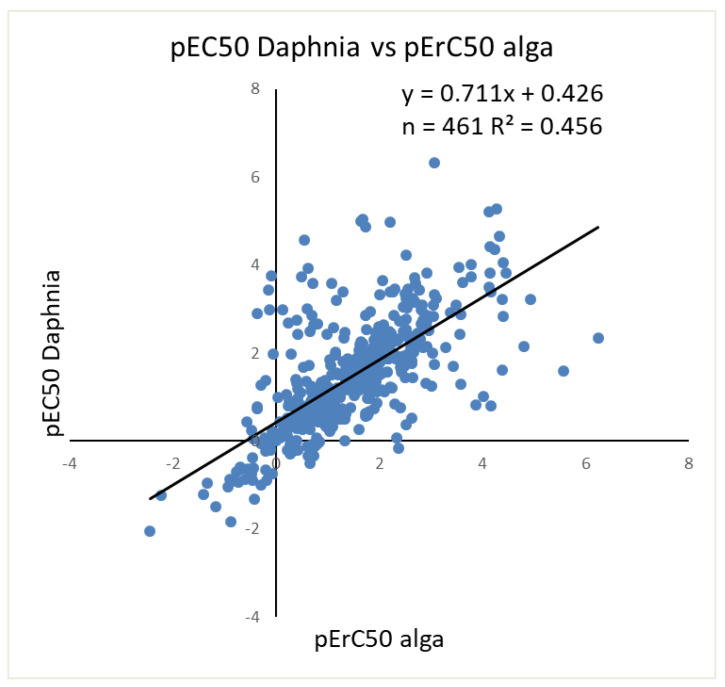
Correlations between the toxicity endpoints.

The data for the toxicity endpoints of species on different trophic levels allow for investigation of correlations between the different toxicities. The correlations between the experimental toxicity towards fathead minnow, *Daphnia magna* (immobilization) and *Raphidocelis subcapitata* are presented in Figure 1. The compounds common for zebrafish and the other endpoints were less than 50; therefore, correlations with zebrafish embryo toxicity are not presented. The correlation coefficients, although moderate (R^2^ in the range of 0.46 to 0.65), indicate positive associations across the data; however, they also indicate some differences, implying that the sets are not fully interchangeable, and any of them provide a unique source for data modeling for the particular endpoint and organisms.

Table 3 summarizes the molecular descriptors used to describe compounds’ structures in the RFR and RFC models.

The structural descriptors listed in Table 3 can be roughly grouped as follows:

Lipophilicity: LogP (octanol–water partition coefficient) calculated by two different methods (XlogP [62,63] and CrippenLogP [64]) appeared as a significant parameter in the obtained models for alga. CrippenLogP is an additive method based on summing contributions from individual atoms or specific molecular fragments; XlogP is an atom-additive method which includes neighboring atom contributions and correction factors.

H-bonding and polarity: nHBAcc3 represents the number of H-bond acceptors. TopoPSA is a measure of the polar surface area. H-bonding potential and polarity reflect the interactions with biological molecules and the water solubility. Molar refractivity (CrippenMR) describes the molecular polarizability. nN, nX and nT6HRing parameters may affect the ionization, polarity and reactivity of the compounds. nAtomP, nBondsD, and nBondsD2 indicate the unsaturation of the molecules.

Steric descriptors: These include descriptors such as MW (molecular weight), RotBtFrac (flexibility), fragC (describing structural complexity), topoDiameter (molecular size and shape).

Electrotopological descriptors [65]: The descriptors in the models, which are from the group of the Electrotopological state (E-state) descriptors, reflect the electron distribution and molecular reactivity. gTopoChargeI reflects the electronic charge distribution and molecular connectivity. Minimum electron E-state (gmin) is related to the presence of electronegative groups. Some of the E-state descriptors are related to the H-bonding potential and polarity of the molecules (minHBa, maxHBd, E-states on -OH, =O, -NH2, groups (minHsOH, SdO, nsNH2)) and are relevant for molecular interactions, as well as reactivity. Minimum and maximum E-State values for specific atom types (minsCH3, minaasC, maxaasC, maxsCH3, MAXDP) are related to the highest electronic influence of the corresponding functional groups (:C:-, -CH3) and compound electrophilicity. Minimum and maximum E-State values for specific hydrogen types (minHdsCH, maxHother) reflect the least reactive potential of these groups. The sum of E-State values for =CH, :CH: (SdsCH, SHaaCH) represents the overall contribution of these groups to molecular reactivity.

### 3.2. Random Forest Regression Models

The obtained RFR models for two of the studied organisms, zebrafish and fathead minnow, are presented in Table 4. The mean value and the minimum and maximum values (shown in brackets) from 10 runs are reported.

The best models observed included five to eight descriptors; increasing the number of descriptors resulted in models with comparable or worse statistical parameters.

RFR models with R^2^_oob and test set R^2^ greater than 0.6 were obtained only for zebrafish embryo and fathead minnow. The RFR models for *Raphidocelis subcapitata* and *Daphnia magna* had lower statistical performance (R^2^ values in the range 0.5–0.6), and these models were excluded from the further analysis [66].

**Figure 2:**
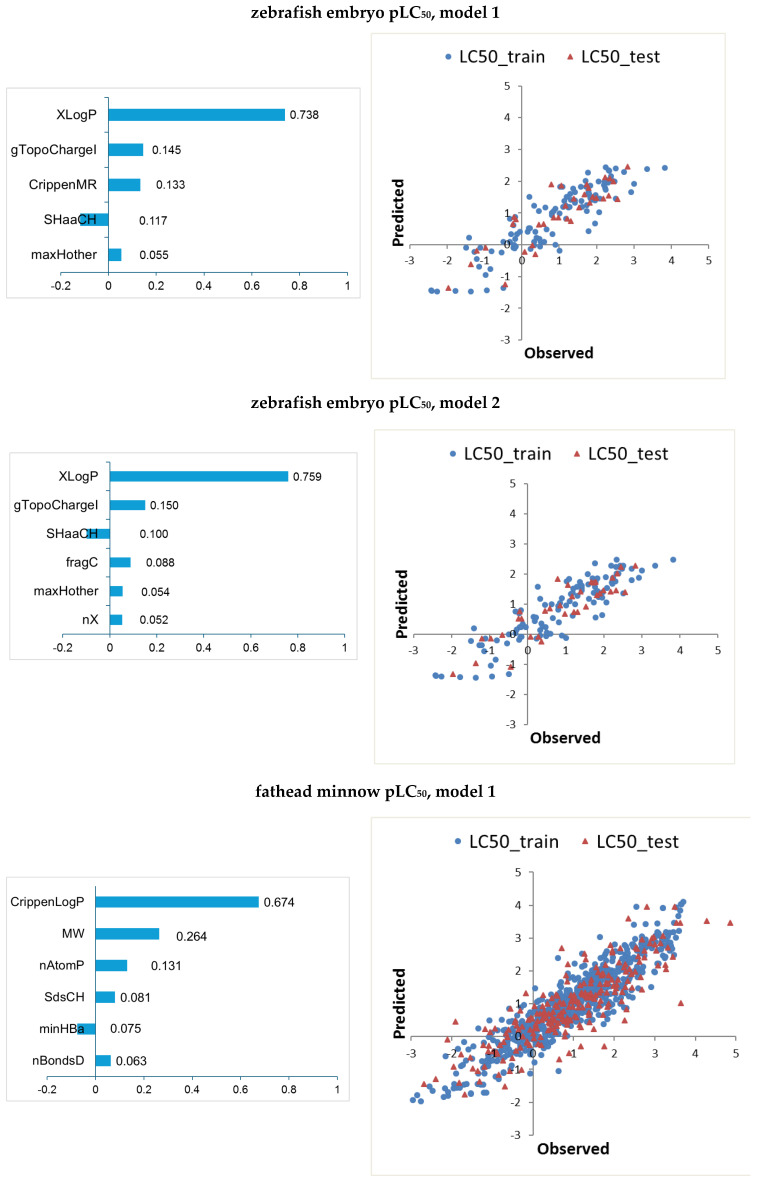

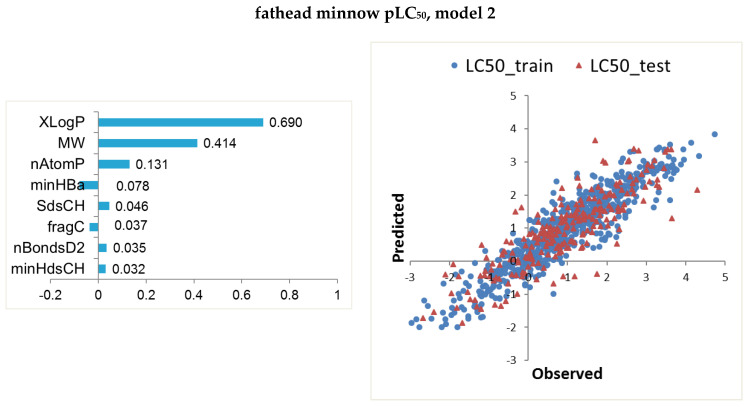
Descriptor SHAP values (**left** panel) and plots of predicted vs. observed toxicity values (**right** panel) for the zebrafish and fathead minnow QSAR models.

Figure 2 gives more details on the derived QSAR models. In the left panel the descriptors and their corresponding average absolute SHAP values are presented. Higher absolute values mean stronger influence of the descriptor on the predicted values. The direction of the corresponding descriptor bar indicates whether the increase in the descriptor value increases (positive direction) or decreases (negative directions) the toxicity. The predicted vs observed values of the models are presented in the right panel of Figure 2.

For all models, the R^2^_adj_ values are very close to the R^2^ values, which confirms the relevance of descriptor inclusion in the models. Very similar values were obtained for the LOO (Q^2^) and OOB (R^2^_oob) statistics in accordance with [57].

CCC values greater than 0.80 suggest good concordance between the observed and predicted toxicity values [67]. All models reported in Table 4 have CCC values exceeding this threshold.

### 3.3. Random Forest Classification Models

The collected datasets include different types of chemicals, pesticides, pharmaceuticals and industrial chemicals tested in different laboratories, which prevents us from obtaining regression QSAR models. Therefore, classification models were constructed to estimate the toxicity towards the alga and the *Daphnia* immobilization effect. The classification models are more straightforward for users as they provide direct information of whether a given compound is toxic or not.

The compounds were classified into three classes (Toxic, Harmful, and Non-toxic) corresponding to the EU chemical hazard categories (see Section 2).

**Figure 3:**
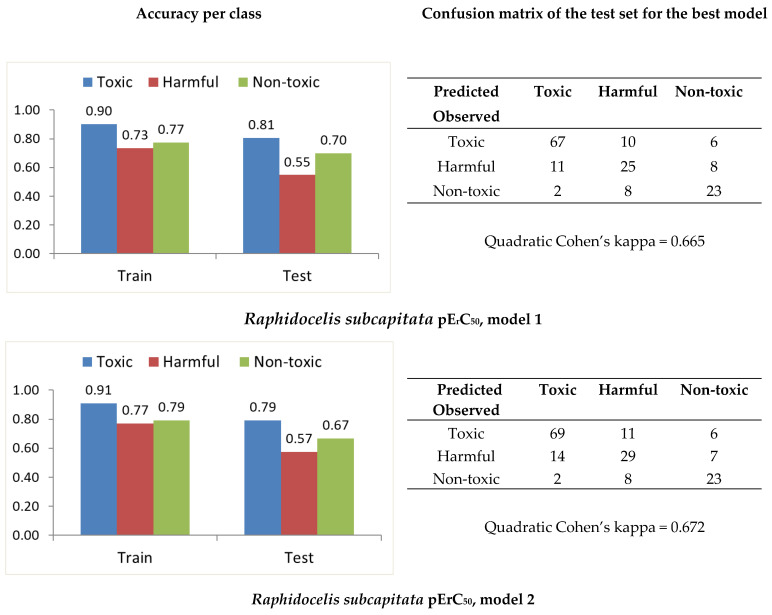

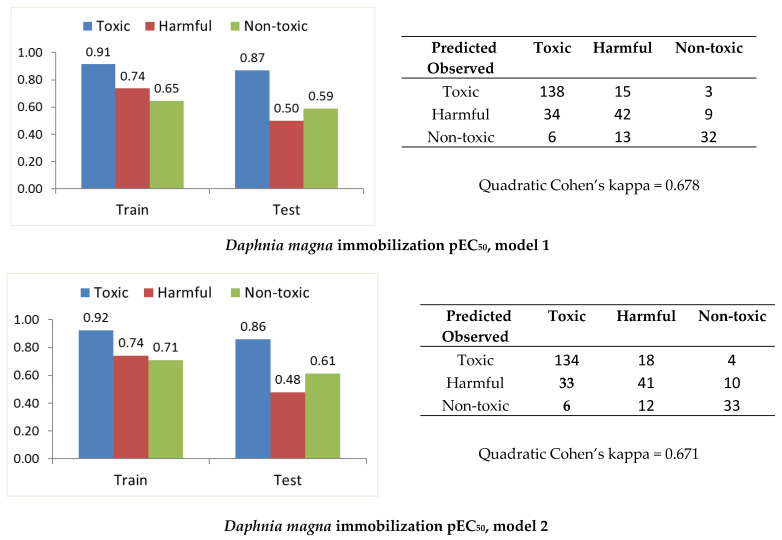
Classification results: Accuracy per class (**left** panel) and confusion matrices of the best run (**right** panel).

The classification models and the corresponding accuracy of classification obtained by chance alone are presented in Table 5 and Figure 3.

The weighted Cohen’s kappa (qCK) statistics reflect the agreement between two samples classified in more than two scaled classes. It incorporates the possibility of the agreement occurring by chance, and also takes into account the distance between the sample values, with larger differences resulting in smaller qCK values [61]. Values above 0.6 indicate substantial agreement between the samples [68]. Our models (Table 5) have values of qCK above 0.6 for both the training and the test sets.

### 3.4. QSAAR Models

**Figure 4:**
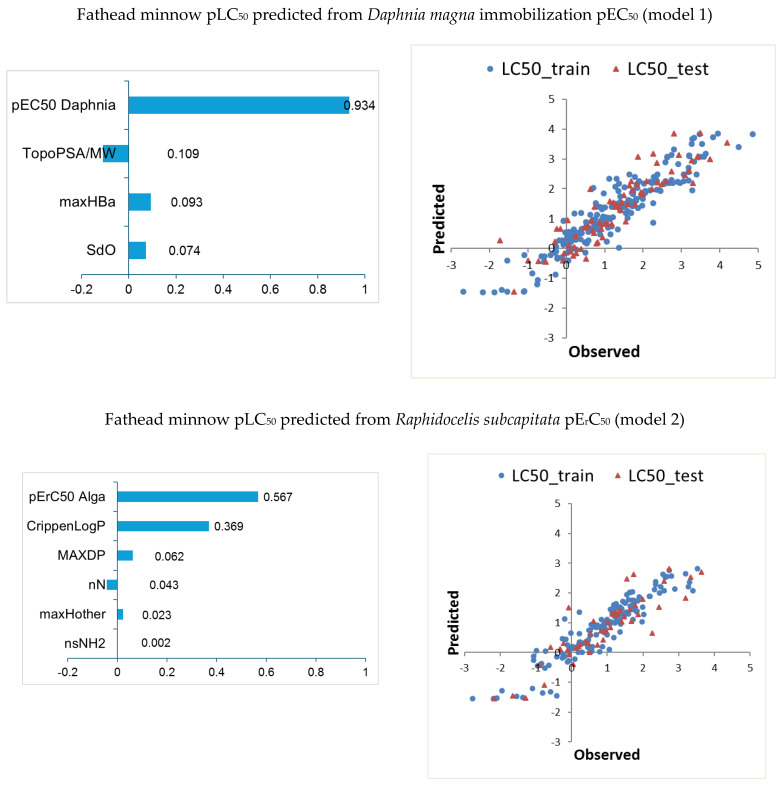
QSAAR results: Independent variables in the models and their corresponding average absolute SHAP values (**left** panels); predicted vs observed toxicity values (**right** panel).

QSAAR models were explored to predict toxicity towards a higher species using toxicity towards lower species and chemical structure descriptors. Detailed structural and toxicological data for QSAAR modeling are presented in the Supplementary QSAAR Data File (File S2). The QSAAR models are presented in Table 6 and Figure 4.

An analysis of the prediction residuals in the RFR models (Table 4 and Table 6) is presented in the Supplementary statistical data file (File S3), Figure S1. Plots of the residuals versus predicted values show a random distribution of residuals around the zero line, which indicates an absence of systematic bias in the model predictions.

## 4. Discussion

### 4.1. Models’ Development

One of the main objectives of the present study was to compile large, curated datasets covering multiple aquatic trophic levels, including four representative aquatic organisms. The datasets were assembled following strict data curation procedures to ensure consistency, reliability, and suitability for QSAR modeling (see Section 2). Data were collected from multiple sources, including different testing laboratories, to maximize the coverage of available information and ensure adequate representation of both chemical structure and toxicity endpoints, in accordance with established modeling standards. At the same time, such broad integration of publicly available data may introduce unwanted variability and experimental noise. To reduce this effect, compounds with toxicity values differing more than three times across sources were excluded from the analysis. In the section below, the models are discussed in order, from organisms at lower trophic levels (algae) to those at higher levels (fish).

The largest datasets used for the development of QSAR models for toxicity towards *Raphidocelis subcapitata* reported in the literature were 271 compounds [66] and 334 compounds [9,12]. Well-structured summaries of the available QSAR models for the alga are presented in Yu [12], Masand et al. [66], and Yu [13]. MLR and SVM techniques were used. The QSAR models demonstrated an R^2^ of the training and test sets of between 0.67 and 0.77. Yu [13] also used 334 compounds and applied RF classification and regression methods. He obtained a model including 22 quantum-chemical descriptors with an R^2^ of the training and test sets of 0.95 and 0.85, respectively. The author also developed two-class classification models by dividing the compounds with a boundary around the median value of the dataset with the same descriptors and obtained a classification accuracy of above 95% for the training set and above 89% for the test set.

For this study, a larger dataset was compiled (695 compounds, 522 in the training set and 173 in the test set) to expand the applicability domain of the models. Further, a three-class classification approach was followed in accordance with the EU regulation classifications for toxic compounds. The best model derived possessed six structural descriptors, enabling better mechanistic interpretation. The descriptors included in the model exhibited low intercorrelation (absolute values of the pair intercorrelation coefficients R of less than 0.7), thereby reducing the risk of overfitting of the model. Although the classification accuracy for ‘harmful’ and ‘non-toxic’ compounds was lower, the models achieved high predictive accuracy for the ‘toxic’ class (90–91% for the training set and 79–81% for the test set, Figure 3). Again, misclassification of the harmful compounds predominantly resulted in their assignment to the ‘toxic’ class (Figure 3), representing a conservative, worst-case scenario.

Experimental data for both mortality and immobilization toxicity towards *Daphnia* are available in the literature. The immobilization endpoint is the standard according to OECD Guideline for the Testing of Chemicals No 202 “*Daphnia* sp. Acute Immobilisation Test” [24]. Further, previous studies such as Rubach et al. [69] have shown that immobilization is a more sensitive endpoint than mortality, as it occurs at lower concentrations and exhibits lower variability across species. Consequently, immobilization can provide a more reliable measure of ecological hazard, whereas mortality effects may not be observed within the standard 48 h exposure period. Therefore, in this study, only data corresponding to the immobilization endpoint were used for modeling.

In the present work, as already discussed, a three-class classification approach was applied, and the class boundaries were set in accordance with the EU classification of toxic compounds towards the aquatic species. In contrast, earlier studies have reported classification models for *Daphnia magna* toxicity based on two toxicity classes [15,70]. He et al. [71] used an approach similar to ours. They reported a three-class model based on data for 639 pesticides (515 training set and 124 test set), categorizing them into compounds with low toxicity (EC_50_ > 100 mg/L), moderate toxicity (in the interval 0.1–100 mg/L) and high toxicity (EC_50_ < 0.1 mg/L). Using structural descriptors and molecular fingerprints, their study reported classification accuracies ranging from 0.794 to 0.848 overall, 0.807 ÷ 0.865 for the high toxicity class, 0.783 ÷ 0.806 for the moderate class, and 0.755 ÷ 0.931 for the low toxicity class. In the present study, a larger, diverse dataset was used (1175 compounds) and overall accuracy of the training and test sets was 81–83% and 71%, respectively (Table 5). The modeling strategy was designed to minimize the risk of underestimating a potential compound toxicity. Thus, the class of toxic compounds was composed by combining the toxic categories of the EU legislation Chronic 1 and Chronic 2 with EC_50_ < 10 mg/L. This class was predicted with high accuracy (91–92% for the training set and 86–87% for the test set). The group of harmful compounds was classified with lower accuracy; however, the misclassifications predominantly resulted in compounds being assigned to the more conservative toxic class (Figure 3), thereby ensuring a worst-case assessment. The non-toxic compounds class was also predicted with lower accuracy; however, this performance can be considered acceptable since from a regulatory perspective, correct identification of toxic compounds is more critical than precise classification of non-toxic ones.

The classification was compared to the classification expected by chance alone. The models showed an improvement over chance of approximately 35–40% for the training set and 26% for the test set, confirming the robustness and predictive value of the model.

Concerning the fish species, RFR QSAR models with good statistical performance were obtained. Furthermore, the narrow ranges observed across the 10 runs for each model indicate high model stability.

Few reports on QSAR models for toxicity to zebrafish embryo are found in the literature, and they include different endpoints (developmental toxicity, lethality, non-hatching, and malformations [72,73]) and different exposure times [7,74]. Toxicity estimated with 96 h LC_50_ values was investigated by Liu et al. [75], who used 68 compounds (54 in the training set and 14 in the test set) and norm-index descriptors of the chemical structure. They obtained a model with R^2^ values of 0.91 (training set) and 0.92 (test set). In the present study, a larger dataset (155 compounds) was used, and the achieved R^2^ was 0.77 ÷ 0.79 for the training set and 0.73 ÷ 0.76 for the test set. The zebrafish dataset remians relatively small, particularly compared to the other datasets included in this study, which may limit the generalizability of the QSAR analysis. Nevertheless, the developed QSAR models can provide useful insights by identifying potential molecular descriptors associated with toxicity trends and may support preliminary screening and priority-setting efforts.

Numerous literature sources report QSAR models for the toxicity of large datasets towards fathead minnow; comprehensive summaries of these models can be found in Cassotti et al. [76], Wu et al. [40] and Wang and Chen [16]. Those studies utilized datasets of varying sizes (from less than 100 [10] up to 963 compounds [39]) and diverse statistical approaches, such as linear regression, partial least squares, neural networks, and k-nearest neighbors. Wu et al. [40] applied MLR to 963 compounds and obtained a model with eight descriptors and R^2^ values of 0.704 (training set) and 0.641 (test set). Wang and Chen [16] developed QSAR models using 955 organic compounds with a radial basis function neural network including 56 structural descriptors and obtained R^2^ values of 0.91 ÷ 0.97 (training set) and 0.72 ÷ 0.74 (test set). Cassotti et al. [76] used 726 chemicals with the k nearest neighbors method and obtained models including six structural descriptors and R^2^ values of between 0.62 and 0.73 (training set) and between 0.61 and 0.77 (test set).

The QSAR models for fathead minnow toxicity developed in this study are based on 941 ÷ 944 compounds (713 ÷ 715 in the training set and 225 ÷ 228 in the test). Although these models showed lower performance compared to those of Wang and Chen [16] for the training set, they demonstrate similar predictive power for the test set (R^2^ between 0.83 and 0.85 for the training set and R^2^ between 0.71 and 0.72 for the test set, Table 4). Notably, our models achieve this while utilizing a significantly smaller number of structural descriptors, allowing for clear mechanistic interpretation.

For each fish toxicity dataset, the two best-performing models are reported, based on partially different sets of structural descriptors. The models are intended to be applied in a consensus manner, i.e., by averaging their predictions. This approach is based on the assumption that the inclusion of diverse structural information across models may capture complementary molecular features governing toxicity, thereby improving overall predictive reliability.

### 4.2. Applicability Domain

Since the datasets include compounds from diverse chemical classes, leverage hat statistics was used to identify compounds with high structural difference from the remaining dataset. These compounds were excluded from the training sets of the models. The leverage analysis was performed individually for each model, based on the specific set of structural descriptors included. In addition, the outliers falling outside the model’s target space were also identified by high prediction residuals (more than 2.5σ, see Section 2) and were excluded from the training sets, thus ensuring model robustness. The compounds from the test sets with high leverages in relation to the descriptor space of the training set, were excluded from the tests sets, as they fell outside of the applicability domain of the training sets.

For *Raphidocelis subcapitata,* the number of compounds excluded from the training set were 19 (model 1, Table 5) and 5 (model 2, Table 5); 13 (model 1, Table 5) and 4 (model 2, Table 5) compounds were excluded from the test set. Model 2 had fewer compounds excluded as leverage and target outliers. For the *Daphnia magna* models, the compounds excluded were between three and eight for the training sets and between one and two for the test sets. Seven and eight compounds were excluded from the training set, and three and four compounds were excluded from the test set of the two models for zebrafish toxicity. For fathead minnow, there were more excluded compounds (44 ÷ 46 for the training set and 29 ÷ 32 for the test set). The above numbers were obtained while adopting a weaker criterion for leverage exclusion (compounds with leverage value smaller than 3(p + 1)/n (where p is the number of model variables plus one, and n is the number of the chemicals in the set)), while some authors indicate a threshold value of 3p/n or 2p/n. In the current study the value of 3(p + 1)/n is adopted in order to have a broader chemical space domain of the training set.

### 4.3. Comparison Among the Models

The RFR QSAR models for the same endpoint derived with different sets of descriptors show comparable results (Table 4, Figure 2). In case of the zebrafish models, two descriptors that appear in both models—CrippenlogP and TopoPSA—point to importance of lipophilic/hydrophilic properties for the toxic effects of chemicals. For the fathead minnow models, in addition to the lipophilicity descriptors, the size (MW and nAtomP) and acceptor H-bonding (minHBa) descriptors have defined roles. For both species, the lipophilicity, although presented by different descriptors (CrippenlogP and XlogP showing the highest SHAP values), appears to be the most essential structural property of the compounds with regard to the observed toxic effects. In the event of comparable statistical parameters and following the rule of Occam’s razor, the QSAR models with smaller numbers of descriptors have to be preferred; however, using both models would allow us to achieve consensus predictions. It is worth noting that the model descriptors are mechanistically meaningful and easy to calculate.

Similarly to the RFR models, the lipophilic properties of the compounds are also persistently present in the classification models. In the case of the *Raphidocelis subcapitata* endpoint, the descriptors of the best two models almost overlap; for the *Daphnia magna* endpoint, the descriptors are more diverse. The models rely on a limited number (six descriptors per model) of meaningful descriptors.

### 4.4. Mechanistic Interpretation of the Structural Descriptors

The structural descriptors with influence on the toxicity, according to the derived models, are related to the molecular lipophilicity, the presence of hydrogen bond (H-bond) acceptors and polar groups, the presence of unsaturated substitutes, and the size and branching of the molecules.

XlogP and CrippenLogP appeared to be significant parameters in the obtained models for alga. The two descriptors intercorrelate with R = 0.869 for the algal dataset and R = 0.867 for the fathead minnow set. These correlations indicate some differences in the calculated CrippenLogP and XlogP values for the datasets, presumably due to the datasets and algorithms implemented, and thus, both descriptors can be used when considering the development of toxicity prediction models.

As RFR is a non-linear approach, a straightforward interpretation of the influence of a given descriptor on the predicted toxicity endpoint is challenging. However, an insight into the descriptors’ importance for the model may be obtained by using the SHAP values (see Section 2). The predicted value of a compound is equal to the sum of the average prediction value for the model and the descriptor SHAP values for the compound. Thus, a greater SHAP value for a given descriptor indicates that this descriptor contributes to a greater predicted value for the compound, and vice versa. The importance of a given descriptor in the model can be assessed by the absolute SHAP values averaged over all compounds. A larger averaged absolute SHAP value corresponds to a greater contribution of the corresponding descriptor to the model prediction.

Because a greater SHAP value for a given descriptor results in a greater predicted value of the endpoint, an idea for the direction of the descriptor influence on the endpoint may be given by the sign correlation between the descriptor values and its SHAP values. Positive correlation between the descriptor and the SHAP values would indicate that increasing the descriptor value results in increasing the predicted endpoint, and vice versa.

The descriptors in the models with the highest positive contribution to the toxicity are related to the compounds’ lipophilicity (CrippenLogP, XlogP) and molecular size (MW, topoPSA), indicating that more lipophilic and bulk molecules are more toxic. The increase in H-bond acceptors and polar groups generally decreases the toxicity, in accordance with the results of Khan and Roy [9]. The molecular complexity (fragC) may have a small negative influence on the toxicity (model 2 for fathead minnow). The toxicity increases with increases in the number of atoms in the largest π-system (nAtomP) and the number of double bonds (nBondsD, nBondsD2), reflecting increased unsaturation of the molecule.

### 4.5. Interspecies Correlations and QSAAR Models

In this study, the intercorrelations between toxicity endpoints for different aquatic species were systematically investigated (Figure 1). Although general trends of positive association can be observed, the intercorrelation coefficients are moderate rather than strong (R^2^ between 0.456 and 0.650). The best correlation was observed between *Daphnia magna* and fathead minnow toxicity. These moderate correlations indicate that while individual endpoints are not directly interchangeable, they may still provide complementary information for cross-species toxicity prediction.

Building on this, QSAAR models were developed to predict toxicity to fathead minnow by integrating toxicity data from species at lower trophic levels with molecular structural descriptors. The best models obtained had two structural descriptors. The developed QSAAR models demonstrated good statistical performance with R^2^ values of 0.830 ÷ 0.878 for the training sets and 0.728 ÷ 0.778 for the external test sets. The structural descriptors included in the models are related to compound lipophilicity (CrippenLogP), H-bonding potential (maxHBa, nN, SdO, nsNH2), basicity and polarity (nN, nsNH2, SdO, TopoPSA/MW), and reactivity (nN, MAXDP, maxHother).

## 5. Conclusions

In the present work, large and structurally diverse, carefully curated datasets for alga (*Raphidocelis subcapitata*), (crustacean) *Daphnia magna*, and fish (zebrafish embryo and fathead minnow) toxicity were collected and used for the development of random forest regression and classification QSAR models. For *Raphidocelis subcapitata* and *Daphnia magna*, classification models were developed by dividing the compounds into three classes (toxic, harmful and non-toxic) in accordance with the EU classification of compounds that are toxic towards aquatic species. The group of toxic compounds was classified with high accuracy (90 ÷ 94% for the training sets, and 80 ÷ 88% for the test sets), demonstrating practical applicability for chemical hazard assessment and regulatory screening. For fish species, robust regression models for zebrafish embryo and fathead minnow toxicity were developed, showing strong statistical performance. These models were based on structurally meaningful descriptors with low intercorrelation (R < 0.7), ensuring reduced redundancy and improved interpretability. The included descriptors capture lipophilicity, polarity, molecular complexity and reactivity, demonstrating their potential to generalize across the modeling of structurally diverse compounds. The correlations between the toxicity endpoints were investigated, and QSAAR models for predicting fathead minnow toxicity from *Raphidocelis subcapitata* and *Daphnia magna* toxicities and structural descriptors were obtained. These models highlight that the combination of lower trophic level toxicity endpoints with structural information can serve as an effective surrogate for estimating toxicity in organisms of higher trophic levels such as fish. The models are in the process of implementation in a freely accessible web-based platform for toxicity prediction, CompuTox Predictor [77].

## Figures and Tables

**Table 1:** Summary of the collected datasets used for QSAR modeling.

Toxicity Endpoint	Number of Training Set Compounds	Number of Test Set Compounds	Toxicity Range (Negative Decimal Logarithm in Units mmol/L)	Toxicity Mean	Sources
*Raphidocelis subcapitata* (alga) E_r_C_50_ of growth rate at 72 h exposure	522	173	−2.045 to 6.834	1.510	ECOTOX [8], EFSA [29], QSAR Toolbox [30], Arouja et al. [31], Gramatica et al. [32], Arouja et al. [33], Singh et al. [34], Sangion and Gramatica [10].
*Daphnia magna* EC_50_ for immobilization at 48 h exposure	882	293	−2.630 to 7.391	1.674	ECOTOX [8], EFSA [29], QSAR Toolbox [30], VEGA [35], Cassani et al. [36], Sangion and Gramatica [10], Khan and Roy [9], Furuhama et al. [19].
*Danio rerio* (zebrafish) embryo LC_50_ at 96 h exposure	117	38	−2.429 to 4.083	0.945	ECOTOX [8], Ali et al. [37], Klüver et al. [38].
*Pimephales promelas* (fish fathead minnow) LC_50_ at 96 h exposure	758	251	−2.965 to 6.890	1.062	ECOTOX [8], EFSA [29], QSAR Toolbox [30], Papa et al. [39], Wang and Chen [16], Wu et al. [40], Sangion and Gramatica [10], Munkittrick et al. [41], Austin et al. [42], Cronin et al. [43], Ren et al. [44], Sinks and Schultz [45], Bearden and Schultz [46], Javorska and Schultz [47], Javorska et al. [48], Schultz et al. [49], Wayne Schultz et al. [50].

**Table 2:** Classification boundaries for the three-class toxicity classification.

Class	Toxic	Harmful	Non-Toxic
Boundary (mg/L)	<10	≥10 and <100	≥100
Boundary (mmol/L)	<0.0439	≥0.0439 and <0.439	≥0.439

**Table 3:** Descriptors used in the RFR and RFC models.

Descriptor Abbreviation	Description
CrippenLogP	Crippen’s LogP
CrippenMR	Crippen’s molar refractivity
fragC	Complexity of a system
gTopoChargeI	Global topological charge index
gmin	Minimum E-State
maxaasC	Maximum atom-type E-State: :C:-
MAXDP	Maximum positive intrinsic state difference in the molecule
maxHBa	Maximum E-States for (strong) hydrogen bond acceptors
maxHBd	Maximum E-States for (strong) hydrogen bond donors
maxHother	Maximum atom-type H E-State, H on :CH:, =CH2 or =CH-
maxsCH3	Maximum atom-type E-State: -CH3
minaasC	Minimum atom-type E-State: :C:-
minHBa	Minimum E-States for (strong) hydrogen bond acceptors
minHdsCH	Minimum atom-type H E-State: =CH-
minHsOH	Minimum atom-type H E-State: -OH
minsCH3	Minimum atom-type E-State: -CH3
MW	Molecular weight
nHBAcc3	Number of hydrogen bond acceptors
nAtomP	Number of atoms in the largest pi system
nBondsD	Number of double bonds
nBondsD2	Number of double bonds, excluding double bonds in aromatic rings
nN	Number of nitrogen atoms
nT6HRing	Number of six-membered rings (includes fused rings) with heteroatoms
nX	Number of halogen atoms
nsNH2	Count of atom-type E-State: -NH2
RotBtFrac	Fraction of rotatable bonds, including terminal bonds
SdO	Sum of atom-type E-State: =O
SdsCH	Sum of atom-type E-State: =CH-
SHaaCH	Sum of atom-type H E-State: :CH:
topoDiameter	Topological diameter (maximum atom eccentricity)
TopoPSA/MW	Topological polar surface area divided by the molecular weight
XLogP	XLogP

**Table 4:** Statistical parameters of the QSAR models for zebrafish and fathead minnow derived from different sets of descriptors. The statistics from 10 RFR runs for each model are presented—mean values and minimum and maximum values (shown in brackets; the statistical parameters are described in the Supplementary statistical data file (File S3)).

Model No.	Endpoint	Descriptors	Training Set						Test Set			
			n	R^2^	R^2^_adj_	SEE	Q^2^	R^2^_oob	n	R^2^	SEE	CCC
1	zebrafish embryo pLC_50_	CrippenMR, gTopoChargeI, maxHother, SHaaCH, XlogP	110	0.783 (0.780 ÷ 0.786)	0.772 (0.770 ÷ 0.777)	0.625 (0.621 ÷ 0.629)	0.556 (0.552 ÷ 0.560	0.559 (0.554 ÷ 0.563)	35	0.738 (0.731 ÷ 0.743)	0.678 (0.671 ÷ 0.687)	0.838 (0.832 ÷ 0.840)
2	zebrafish embryo pLC_50_	fragC, gTopoChargeI, maxHother, nX, SHaaCH, XlogP	109	0.781 (0.767 ÷ 0.786	0.768 (0.754 ÷ 0.773	0.637 (0.629 ÷ 0.659)	0.551 (0.520 ÷ 0.561)	0.551 (0.518 ÷ 0.568)	36	0.758 (0.746 ÷ 0.765)	0.668 (0.658 ÷ 0.683)	0.845 (0.837 ÷ 0.850)
1	fathead minnow pLC_50_	CrippenLogP, minHBa, MW, nAtomP, nBondsD, SdsCH	715	0.832 (0.830 ÷ 0.835)	0.831 (0.828 ÷ 0.834)	0.553 (0.546 ÷ 0.557)	0.678 (0.674 ÷ 0.684)	0.678 (0.672 ÷ 0.684)	228	0.716 (0.710 ÷ 0.720)	0.735 (0.730 ÷ 0.744)	0.833 (0.831 ÷ 0.835)
2	fathead minnow pLC_50_	fragC, minHBa, minHdsCH, MW, nAtomP, nBondsD2, SdsCH, XLogP	713	0.851 (0.849 ÷ 0.854)	0.850 (0.847 ÷ 0.852)	0.516 (0.513 ÷ 0.521)	0.696 (0.691 ÷ 0.699)	0.696 (0.690 ÷ 0.701)	225	0.719 (0.717 ÷ 0.722)	0.721 (0.718 ÷ 0.724)	0.838 (0.836 ÷ 0.839)

**Table 5:** Statistical parameters of the QSAR classification models for *Raphidocelis subcapitata* and *Daphnia magna* derived from the best set of descriptors (the statistical parameters are described in the Supplementary statistical data file (File S3)).

Model No.	Endpoint	Descriptors	Accuracy by Chance	Training Set				Test Set		
				n	Acc	Acc_oob	qCK	n	Acc	qCK
1	*Raphidocelis subcapitata* pE_r_C_50_	CrippenLogP, maxaasC, minHBa, minHsOH, nHBAcc3, topoDiameter	37.6	503	82.7 (82.3 ÷ 83.1)	68.1 (67.4 ÷ 68.8)	0.753 (0.743 ÷ 0.769	160	71.4 (70.0 ÷ 71.9)	0.653 (0.637 ÷ 0.665)
2	*Raphidocelis subcapitata* pE_r_C_50_	CrippenLogP, maxsCH3, minHBa, minHsOH, RotBtFrac, topoDiameter	37.9	517	84.5 (83.9 ÷ 85.1)	67.9 (67.1 ÷ 68.7)	0.772 (0.765 ÷ 0.777	169	70.3 (69.2 ÷ 71.6)	0.649 (0.627 ÷ 0.672)
1	*Daphnia magna* immobilization pEC_50_	CrippenLogP, maxHBd, minHBa, minHsOH, minsCH3, nT6HRing	45.1	875	81.5 (80.9 ÷ 82.5)	67.9 (67.3 ÷ 68.7)	0.745 (0.738 ÷ 0.757	292	71.3 (70.2 ÷ 72.6)	0.662 (0.650 ÷ 0.678)
2	*Daphnia magna* immobilization pEC_50_	CrippenLogP, gmin, maxHBd, minaasC, minHBa, minsCH3	43.9	874	83.3 (82.2 ÷ 84.2)	68.0 (67.4 ÷ 68.4)	0.771 (0.753 ÷ 0.782)	291	70.6 (69.8 ÷ 71.5)	0.658 (0.641 ÷ 0.671)

**Table 6:** Statistical parameters of the developed QSAAR models.

Model No.	Dependent Endpoint	Independent Endpoint Used in the Equation	Descriptors	Training Set						Test Set			
				n	R^2^	R^2^_adj_	SEE	Q^2^	R^2^_oob	n	R^2^	SEE	CCC
1	fathead minnow pLC_50_	*Daphnia magna* immobilization pEC_50_	maxHBa, SdO, TopoPSA/MW	203	0.875 (0.874 ÷ 0.876)	0.874 (0.871 ÷ 0.879)	0.478 (0.476 ÷ 0.480)	0.773 (0.770 ÷ 0.774)	0.772 (0.769 ÷ 0.777)	71	0.813 (0.811 ÷ 0.814)	0.597 (0.594 ÷ 0.600)	0.898 (0.897 ÷ 0.899)
2	fathead minnow pLC_50_	*Raphidocelis subcapitata* pE_r_C_50_	CrippenLogP, MAXDP, maxHot-her, nN, nsNH2	143	0.854 (0.851 ÷ 0.860)	0.847 (0.845 ÷ 0.853)	0.478 (0.467 ÷ 0.483)	0.720 (0.716 ÷ 0.729)	0.719 (0.712 ÷ 0.733)	48	0.781 (0.774 ÷ 0.792)	0.626 (0.611 ÷ 0.634)	0.872 (0.867 ÷ 0.879)

## Data Availability

The original data used in this study are included in the Supplementary Materials. Further inquiries can be directed to the corresponding authors.

## Supplementary Materials

The following supporting information can be downloaded at https://www.mdpi.com/article/10.3390/toxics14060498/s1, File S1. Supplementary QSAR data. File S2. Supplementary QSAAR data. File S3. Supplementary statistical data. Figure S1. Analysis of residuals of the RFR models (plots of residuals vs predicted values).

## Abbreviations

The following abbreviations are used in this manuscript:

MLR	Multiple linear regression
NN	Neural networks
OOB	Out-of-bag
LOO	Leave-one-out
SEE	Standard error of estimate
CCC	Concordance correlation coefficient
PLS	Partial least squares
QSAR	Quantitative structure–activity relationship
QSAAR	Quantitative structure–activity–activity relationship
RBF	Radial basis function
RFR	Random forest regression
RFC	Random forest classification
SHAP	Shapley additive explanations
SVM	Support vector machine
