# Supplementary material for: QSAR Modeling to Predict Aquatic Toxicity Across Multiple Species
Source: Toxics. 2026 Jun 7;14(6):498. doi: 10.3390/toxics14060498 (PMC13307692; doi:10.3390/toxics14060498)
Supplement: Supplementary file 1 [file toxics-14-00498-s001.zip › File S3. Supplementary statistical data.pdf]

toxics

MDPI

Statistical parameters for model assessment

The goodness-of-fit of the RFR models was assessed using the coefficients of determination (R^2) and the standard error of estimate (SEE), where y_{pred} represents the predicted values of the target, y_{obs} is the experimental values of the target, y_{mean} is the mean of the experimental values, n is the number of observations, and p is the number of independent variables (descriptors) in the model:

$$R^2 = 1 - \frac{\sum(y_{pred} - y_{obs})^2}{\sum(y_{pred} - y_{mean})^2} \quad \text{(S1)}$$

$$SEE = \sqrt{\frac{\sum(y_{pred} - y_{obs})^2}{(n - p - 1)}} \quad \text{(S2)}$$

The adjusted coefficient of determination R^2_{adj} is obtained by dividing the numerator and denominator of the equation (S1) by their corresponding degrees of freedom:

$$R^2_{adj} = 1 - \frac{\sum(y_{pred} - y_{obs})^2/(n - p - 1)}{\sum\left(y_{pred} - y_{mean}\right)^2/(n - 1)} = 1 - (1 - R^2)\frac{n - 1}{n - p - 1} \quad \text{(S3)}$$

The concordance correlation coefficient (CCC) between two continuous variables is calculated using the following formula (R is the correlation coefficient between the two variables, μ_1 and μ_2 are their means, σ_1^2 and σ_2^2 are their variances):

$$CCC = \frac{R\sigma_1\sigma_2}{\sigma_1^2 + \sigma_2^2 + (\mu_1 - \mu_2)^2} \quad \text{(S4)}$$

The accuracy of classification models (Acc) is calculated as follows:

$$Acc(\%) = \frac{number\ of\ correctly\ classified\ compounds}{total\ number\ of\ compounds} * 100 \quad \text{(S5)}$$

The quadratic weighted Cohen's Kappa (qCK) is equal to

$$qCK = 1 - \frac{\sum w_{ij}P_{ij}}{\sum w_{ij}E_{ij}} \quad \text{(S6)}$$

where w_{ij} are quadratic weights proportional to the square of the distances between the classes, P_{ij} are the elements of the confusion matrix, and E_{ij} is the matrix of the expected frequencies.

The percentage of correct classifications is compared to the percentage of cases that would have been correctly classified by chance alone. For n groups of different sizes (g1, g2,..., gn), the expected correct classification is equal to

$$expected\ percent\ correct\ classification\ by\ chance\ alone = \frac{\frac{g1}{n} * g1 + \frac{g2}{n} * g2 + \ldots + \frac{gn}{n} * gn}{n} \quad \text{(S7)}$$

zebrafish embryo pLC$_{50}$, Table 4, Model 1

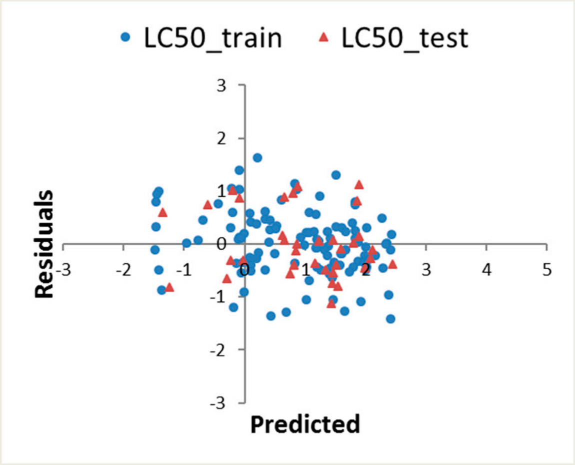

zebrafish embryo pLC$_{50}$, Table 4, Model 2

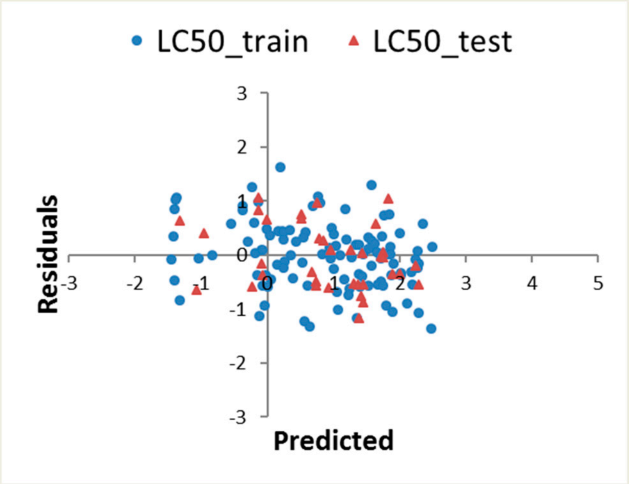

fathead minnow pLC$_{50}$, Table 4, Model 1

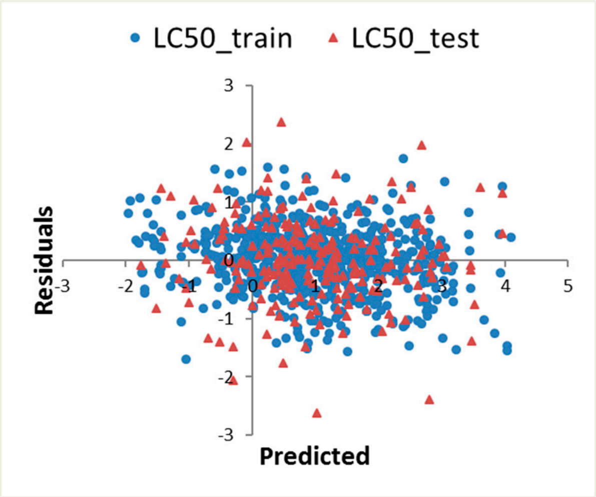

fathead minnow pLC$_{50}$, Table 4, Model 2

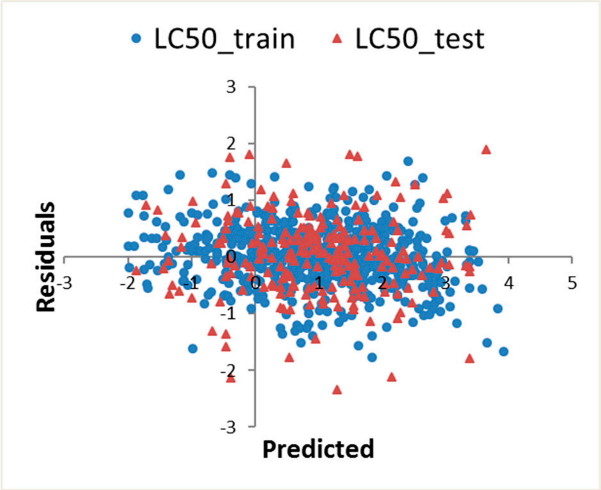

fathead minnow pLC$_{50}$, Table 6, Model 1

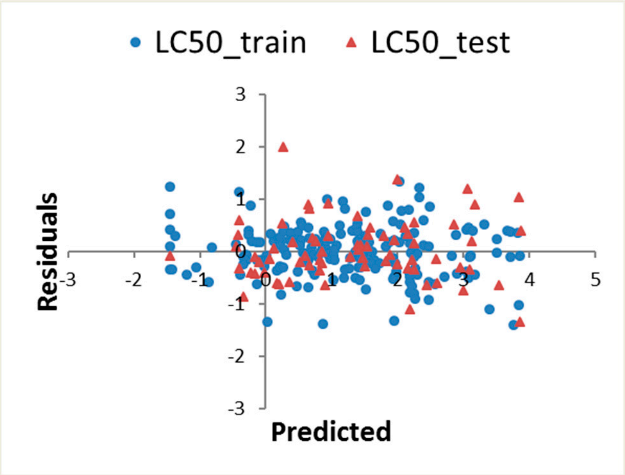

fathead minnow pLC$_{50}$, Table 6, Model 2

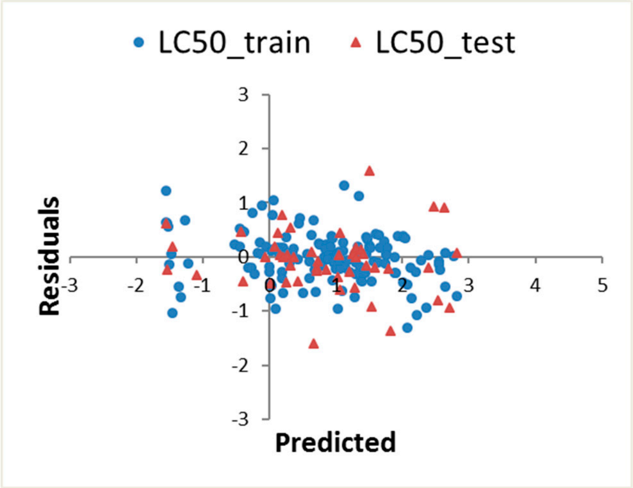

Figure S1. Analysis of residuals of the RFR models (plots of residuals vs predicted values).
